# *Anopheles gambiae s.l.* swarms trapping as a complementary tool against residual malaria transmission in eastern Gambia

**DOI:** 10.1038/s41598-022-21577-7

**Published:** 2022-10-12

**Authors:** Benoît Sessinou Assogba, Salimina Sillah, Kevin O. Opondo, Sheikh Tijan Cham, Muhammed M. Camara, Lamin Jadama, Lamin Camara, Assane Ndiaye, Miriam Wathuo, Musa Jawara, Abdoulaye Diabaté, Jane Achan, Umberto D’Alessandro

**Affiliations:** 1 Disease Control and Elimination Theme, Medical Research Council, Unit The Gambia at London School of Hygiene and Tropical Medicine, PO Box 273, Banjul, The Gambia; 2 Statistic and Bioinformatic Department, Medical Research Council, Unit The Gambia at London School of Hygiene and Tropical Medicine, PO Box 273, Banjul, The Gambia; 3 Institut de Recherche en Science de la Santé/Centre Muraz, BP 545, Bobo-Dioulasso, Burkina Faso; 4 Laboratoire d’Ecologie Vectorielle et Parasitaire, Faculté des Sciences et Techniques, Université Cheikh Anta Diop, Dakar, Sénégal

**Keywords:** Malaria, Entomology, Epidemiology

## Abstract

Malaria remains a major health problem and vector control is an essential approach to decrease its burden, although it is threatened by insecticide resistance. New approaches for vector control are needed. The females of *Anopheles gambiae s.l.* mate once in their life and in the swarms formed by males. Trapping swarms of *Anopheles gambiae s.l.* males is a potential new intervention for vector control, alternative to the use of insecticides, as it would disrupt mating . The proof-of-concept pilot study aiming at investigating swarm trapping as a potential vector control intervention, was carried out in 6 villages as in eastern Gambia. Swarms of *Anopheles gambiae s.l.* were identified and their size, height, and duration determined during the baseline year. Swarm trapping by local volunteers was implemented the following transmission season in 4 villages while the other 2 villages were taken as controls. Entomological outcomes were monitored by Human Landing Catches and Pyrethrum Spray Catches. A cross-sectional survey to determine malaria prevalence was carried out at the peak of the malaria transmission season for two consecutive years. At baseline, 23 swarming sites of *Anopheles gambiae s.l.* were identified. Before the intervention, mean indoor resting density per house and malaria prevalence were similar between control and intervention villages. Following the intervention, *Anopheles gambiae s.l.* indoor resting density was 44% lower in intervention than in control villages (adj IRR: 0.0.56; 95% CI 0.47–0.68); the odds of malaria infections were 68% lower in intervention than in control villages (OR: 0.32; 95% CI 0.11–0.97). Swarm trapping seems to be a promising, community-based vector control intervention that could reduce malaria prevalence by reducing vector density. Such results should be further investigated and confirmed by larger cluster-randomized trials.

## Introduction

Vector-borne diseases represent a considerable health burden in tropical and subtropical regions. Malaria alone, transmitted exclusively by *Anopheles* mosquitoes (Diptera: Culicidae) infected with *Plasmodium* protozoan parasites, caused in 2019 about 229 million cases and 409,000 deaths, most of them in sub-Saharan Africa^1^. Between 2000 and 2015, thanks to the scale up of standard control interventions, the malaria burden decreased substantially in several sub-Saharan countries^1,2^. However, transmission is still ongoing and resistance against antimalarial drugs and insecticides has emerged^3,4^. Moreover, where coverage of standard control interventions is high, residual transmission is maintained by outdoor transmission^5–8^. Therefore, new tools are needed as standard control interventions are unable to further reduce and then interrupt malaria transmission^1^.

Vector control based on the use of insecticides is a key component of malaria control^2^. Besides insecticide resistance, vector control is challenged by vectors feeding and resting outdoor, allowing them to escape standard control interventions^6^. There is growing interest in the use of genetically modified mosquitoes or laboratory-reared male mosquitoes that, by mating with wild female mosquitoes, would reduce or suppress vector populations through several mechanisms, including sterile insect technique and incompatible insect technique^9,10^. One major challenge for their large-scale implementation is the capacity of such laboratory-reared males to successfully compete with wild ones as basic life history traits and mating preference remain poorly understood ^11^. Moreover, mass rearing and negative effects on off-target species should be considered^12^. In Sub-Saharan Africa, the logistics of implementing such novel tools would be particularly challenging given the lack of biomedical industries, ethic community/government acceptance and expertise to monitor its implementation.

Trapping swarms of male mosquitoes may be an alternative approach for vector control. *An. gambiae s.l.* males form swarms daily, at sunset, for 10–30 min and in the same locations for several years^13^; females mate in flight once in their life, stock the sperm in their spermatheca and then lay eggs every two-days after blood feeding^14^. Mass trapping of males during swarming could significantly reduce indoor resting density by reducing mating and thus malaria transmission. However, for its successful deployment, swarms should be easily identifiable and consistently in the same locations as in Burkina Faso, Mali and Benin, where local residents were trained to identify and collect swarms^14,15^. Such an intervention, despite its apparent difficulties, has the advantage of targeting malaria vectors that may be resistant to insecticides. In Burkina Faso, swarms trapping decreased vector density by 80%^16^, although the impact of such intervention on malaria prevalence was not measured. As female mating increases vector’s susceptibility to human malaria parasites^17^, trapping male mosquitoes may reduce malaria transmission also by this mechanism.

## Results

### Characteristics of *Anopheles gambiae s.l.* reproductive swarms in Eastern Gambia

**Figure 1:**
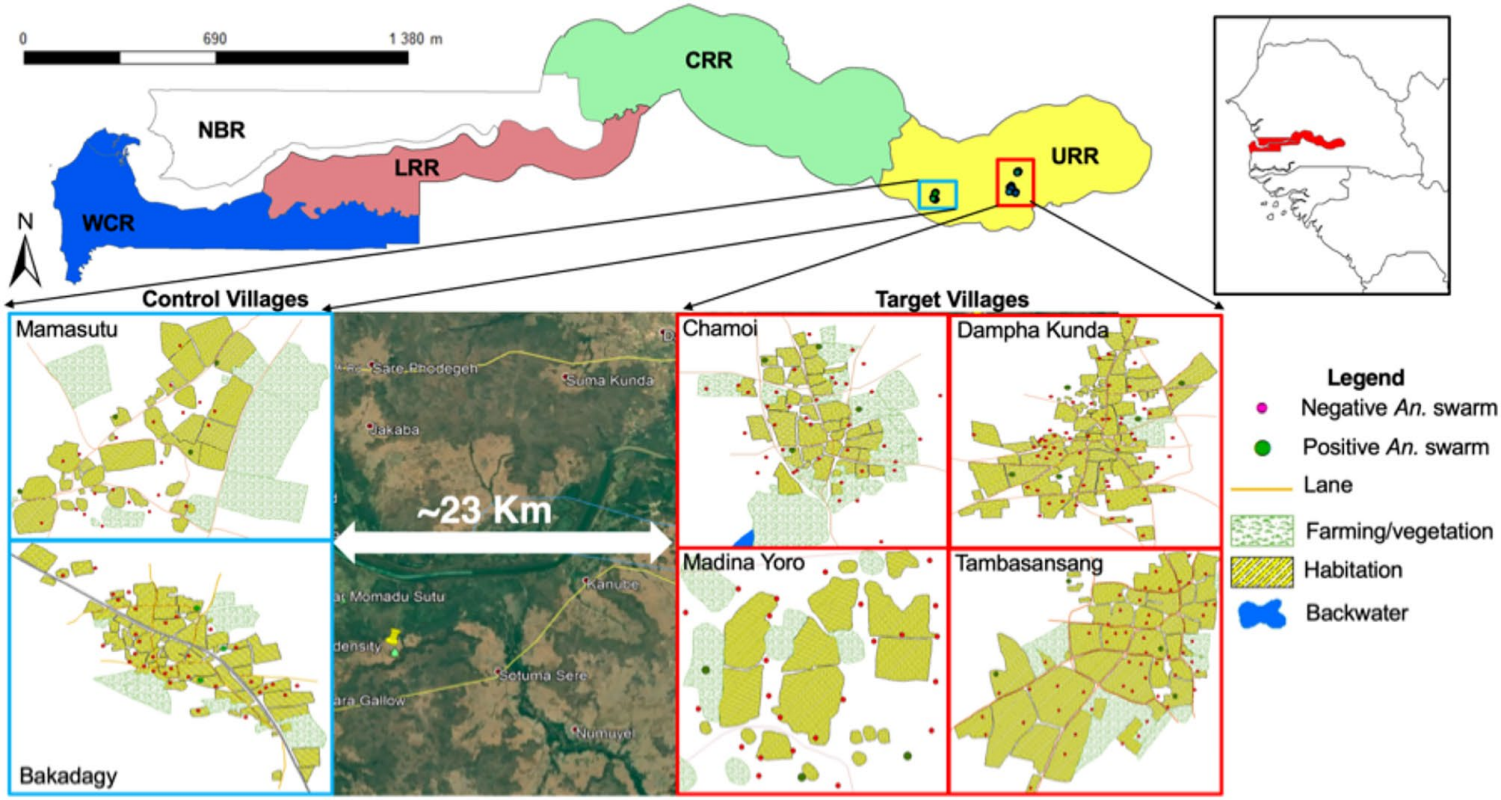
Distribution of *Anopheles gambiae s.l.* reproductive swarms in six villages of eastern Gambia. A The map of The Gambia shows the five administrative regions: WCR (West Coast Region); NBR (North Bank Region), LRR (Lower River Region), CRR (Central River Region) and URR (Upper River Region). The study area is in URR; Blue dots: control villages (Mamasutu and Bakadagy); Red dots: intervention villages (Chamoi, Dampha kunda, Tambasansang and Madina Yoro) the red dots. The control and intervention villages were ~ 23 km apart showing on the satellite image obtained from Google Earth Pro 7.3.4.8642. The green circles correspond to the positive *Anopheles gambiae s.l.* swarming positions and red circles correspond to the negative ones.

**Figure 2:**
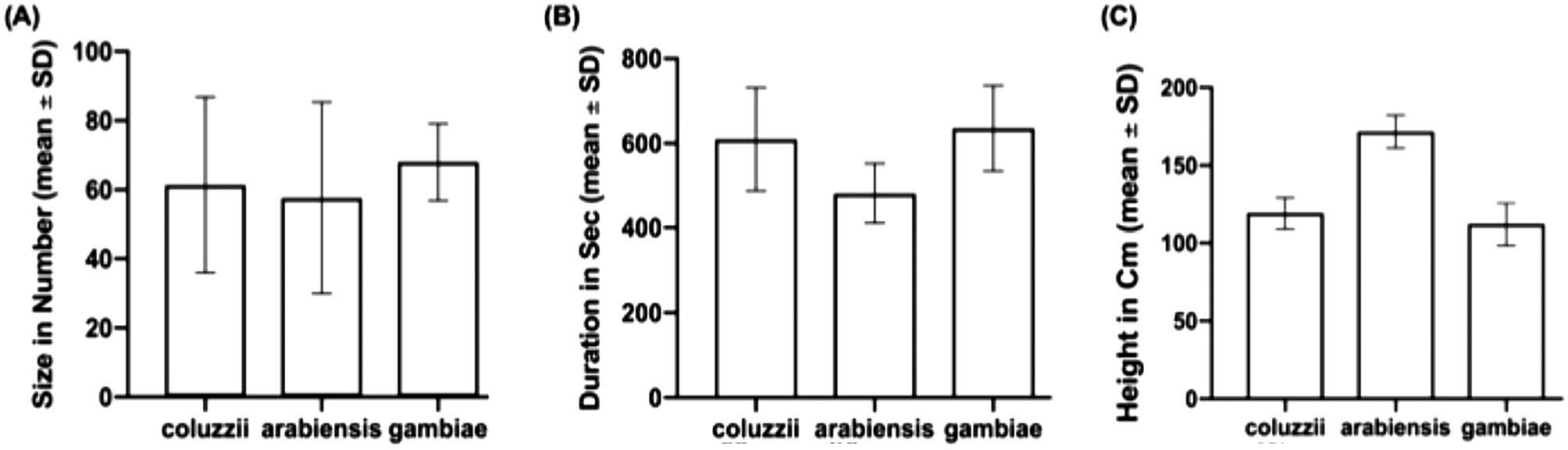
Characteristics of *Anopheles gambiae s.l.* reproductive swarm by species. (**A**) Swarm size; (**B**) Swarming duration; (**C**) Swarming height.

Twenty-three swarming sites of *Anopheles gambiae s.l.*, between 3 and 5 swarms per village, were identified (Table 1), most of them near households (Fig. 1). These swarms usually appeared at the same location a few minutes after sunset, around 18:50. All swarms were species-specific, mostly *An. coluzzii* and *An. arabiensis* (Table 1). Swarm size was 61.34 (standard deviation SD: 25.41), 57.64 (SD: 27.72), and 68 (SD: 11.11) for *An. coluzzii*, *An. arabiensis* and *An. gambiae s.s.*, respectively, with no difference between species (Fig. 2A, p = 0.44). Mean swarm duration in seconds was 609.94 (SD: 121.88), 481.72 (SD: 70.96) and 635.8 (SD: 100.62) for *An. coluzzii*, *An. arabiensis* and *An. gambiae s.s.*, respectively, with *An. arabiensis* having the shorter duration (Fig. 2B, p < 0.0001) while there was no difference between *An. coluzzii* and *An. gambiae s.s.*. Conversely, swarm height (in centimeter) was significantly higher for *An. arabiensis* (171.8 SD: 10.49) than for *An. coluzzii* (119.05 SD: 10.27) and *An. gambiae s.s.* (112 SD: 13.50) (Fig. 2C, p < 0.0001)*.*

**Table 1:** The characteristic of *Anopheles gambiae s.l.* reproductive swarms observed in six villages of Upper River Region (URR) of The Gambia.

Swarm ID	Village	Starting time	Duration (s)	Height (cm)	Size	Specie	Swarm marker
V1SW_02	Chamoi	18:54:00 ± 0:05	549.2 ± 121.17	129 ± 15.17	58 ± 31.38	*An. coluzzii*	Fire wood
V1SW_07	Chamoi	18:54:00 ± 0:03	571 ± 105.57	130 ± 12.25	62.2 ± 25.78	*An. coluzzii*	Bar ground
V1SW_14	Chamoi	18:52:00 ± 0:04	516.4 ± 109.91	121 ± 20.74	40.2 ± 18.67	*An. coluzzii*	Bar ground
V1SW_27	Chamoi	18:52:00 ± 0:07	525.2 ± 108.25	181 ± 5.48	65.2 ± 26.08	*An. arabiensis*	Bar ground
V2SW_17	Dampha Kunda	18:56:00 ± 0:05	597.4 ± 93.95	117 ± 6.71	58.4 ± 23.65	*An. coluzzii*	Fire wood
V2SW_23	Dampha Kunda	18:53:00 ± 0:02	677 ± 111.45	116 ± 8.94	60 ± 26.65	*An. coluzzii*	Bar ground
V2SW_25	Dampha Kunda	18:50:00 ± 0:02	689.2 ± 214.19	118 ± 2.74	68.6 ± 28.50	*An. coluzzii*	Bar ground
V2SW_42	Dampha Kunda	18:53:00 ± 0:04	669.6 ± 197.48	121 ± 7.42	60.6 ± 19.06	*An. coluzzii*	Bar ground
V2SW_56	Dampha Kunda	18:50:00 ± 0:04	478 ± 49.44	176 ± 11.94	51.8 ± 26.02	*An. arabiensis*	Glass
V3SW_06	Tambasansang	18:52:00 ± 0:05	623 ± 39.45	119 ± 7.42	57.8 ± 18.79	*An. coluzzii*	Bar ground
V3SW_25	Tambasansang	18:51:00 ± 0:04	672 ± 91.21	116 ± 5.48	59.4 ± 25.42	*An. coluzzii*	Bar ground
V3SW_33	Tambasansang	18:52:00 ± 0:02	674.6 ± 112.20	117 ± 9.75	55.6 ± 30.40	*An. coluzzii*	Fire wood
V3SW_50	Tambasansang	18:54:00 ± 0:04	468 ± 70.23	170 ± 7.91	51 ± 33.04	*An. arabiensis*	Fire wood
V4SW_01	Madina Yoro	18:54:00 ± 0:03	548.4 ± 115.53	115 ± 10.00	58.6 ± 31.02	*An. coluzzii*	Glass
V4SW_08	Madina Yoro	18:53:00 ± 0:05	608 ± 153.28	113 ± 12.04	49.2 ± 17.98	*An. coluzzii*	Bar ground
V4SW_24	Madina Yoro	18:55:00 ± 0:04	461.4 ± 62.89	168 ± 8.37	35.4 ± 15.13	*An. arabiensis*	Bar ground
V5SW_08	Mamasutu	18:51:00 ± 0:03	476 ± 63.19	164 ± 11.40	84.8 ± 14.96	*An. arabiensis*	Roof
V5SW_12	Mamasutu	18:50:00 ± 0:04	635.8 ± 100.62	112 ± 13.51	68 ± 11.11	*An. gambiae ss*	Bar ground
V5SW_14	Mamasutu	18:53:00 ± 0:05	634.4 ± 95.95	117 ± 6.71	89 ± 27.64	*An. coluzzii*	Bar ground
V5SW_18	Mamasutu	18:51:00 ± 0:04	565.8 ± 78.54	118 ± 8.37	77 ± 33.67	*An. coluzzii*	Fire wood
V6SW_04	Bakadagy	18:53:00 ± 0:02	552.4 ± 120.77	121 ± 5.48	65.6 ± 15.90	*An. coluzzii*	Glass
V6SW_18	Bakadagy	18:54:00 ± 0:04	587.6 ± 90.59	119 ± 11.40	70 ± 27.78	*An. coluzzi*	Fire wood
V6SW_25	Bakadagy	18:50:00 ± 0:06	633 ± 106.28	117 ± 10.95	53 ± 24.79	*An. coluzzii*	Waste

The swarm ID is an identification code attributed to each position where the reproductive swarm has been found. The quantitative variables (starting time, duration, height and size) are the mean ± SD of five different observations. The stating time is GMT + 0. The duration and height are respectively in second and centimetre. The size is the number of *An. gambae s.l.* male attending the swarming event.

### Swarm trapping

Forty-eight swarm collections (twice a week) during the intervention period captured 36,327 male mosquitoes, 30,131 from *An. coluzzii* swarms and 6196 from *An. arabiensis* swarms. The highest number of mosquitoes was collected in September (Supplementary Fig. 1).

### Indoor resting density and other entomological measurements before and after intervention

**Figure 3:**
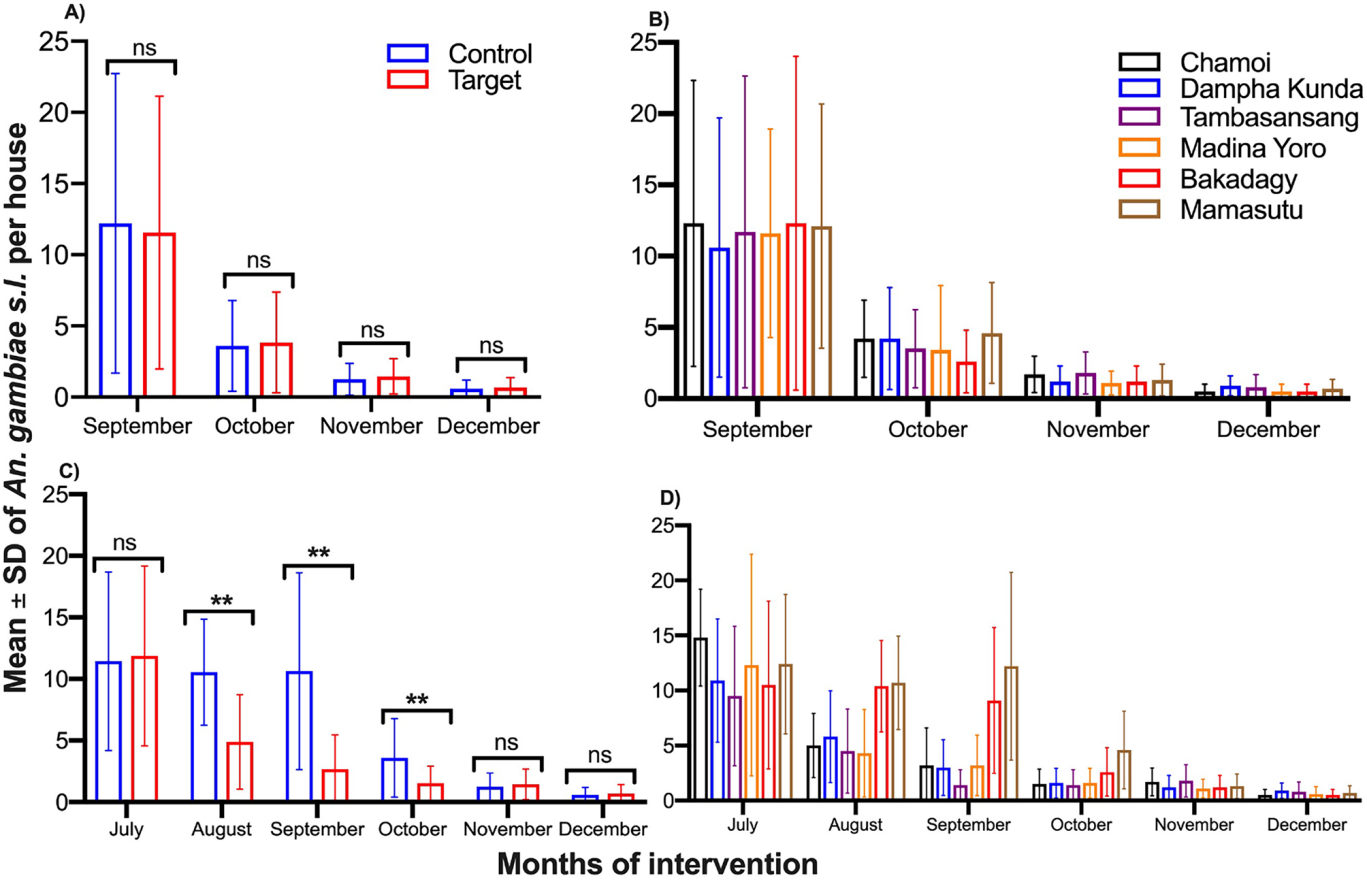
*Anopheles gambiae s.l.* density by study arm and village. Baseline vector density between September and December 2017 by study arm (**A**) and by village (**B**). Vector density during and after intervention(July–December 2018) by study arm (**C**) and by village (**D**).

Before the intervention*,* mean indoor resting density per house was similar between control and intervention villages (adjusted IRR: 1.04; 95% CI 0.80–1.35) (p = 0.964) (Fig. 3A,B, Table 2 and Table S1), and between villages (Fig. 3B). In 2018, mean indoor resting density was significantly lower in intervention than in control villages (adjusted IRR: 0.56; 95% CI 0.47–0.68) (p < 0.001). Such a difference was particularly marked between August and October (Fig. 3C,D and Table 2).

**Table 2:** Results of the unadjusted and adjusted negative binomial regression analyses of *Anopheles gambiae s.l.* density per house compared between control and target arms in 2017 and 2018.

Factors		Unadjusted analysis		Adjusted analysis	
Years	Interventions arms	IRR (95% CI)	p-value	IRR (95% CI)	p-value
2017	Target	Reference	0.964	Reference	0.78
	Control	0.99 (0.68–1.44)		1.04 (0.80–1.35)	
2018	Control	Reference	< 0.001	Reference	< 0.001
	Target	0.61 (0.46–0.80)		0.56 (0.47–0.68)	

IRR (95% CI) correspond to the Incidence rate ratios (IRR) with 95% confidence intervals (95% CI) from unadjusted and adjusted negative binomial regression analyses.

In 2017, a total of 153 *An. gambiae s.l.* were collected by HLC, all of them negative for *P. falciparum* sporozoites. Biting rates ranged between 1 and 1.91 per human/night and were similar between villages.

In 2018, a total of 2560 *An. gambiae s.l.* were collected by HLC, most of them either *An. coluzzii* (52.61%) or *An. arabiensis* (36.95%) (Supplementary Fig. S2A). The biting rate was 10.80 per human/night (95% CI 10.15–11.48) in intervention villages and 15.87 per human/night (95% CI 15.09–16.68) in control villages (p = 0.89, Fig. S2B and Table S2). The sporozoite rate was 0.1% (95% CI 0.02–0.54) in intervention villages and 0.2% (95% CI 0.07–0.56) in control villages (p = 0.9, Supplementary Fig. S2C and Table S3). The EIR was 0.01 (95% CI 0.00–0.06) in the intervention and 0.06 (95% CI 0.01, 0.2) in the control arm (p-value = 0.89, Supplementary Fig. S2D).

### Malaria prevalence

A total of 921 and 892 individuals were included into the cross-sectional survey in 2017 and 2018, respectively (Supplementary Table S4).

**Figure 4:**
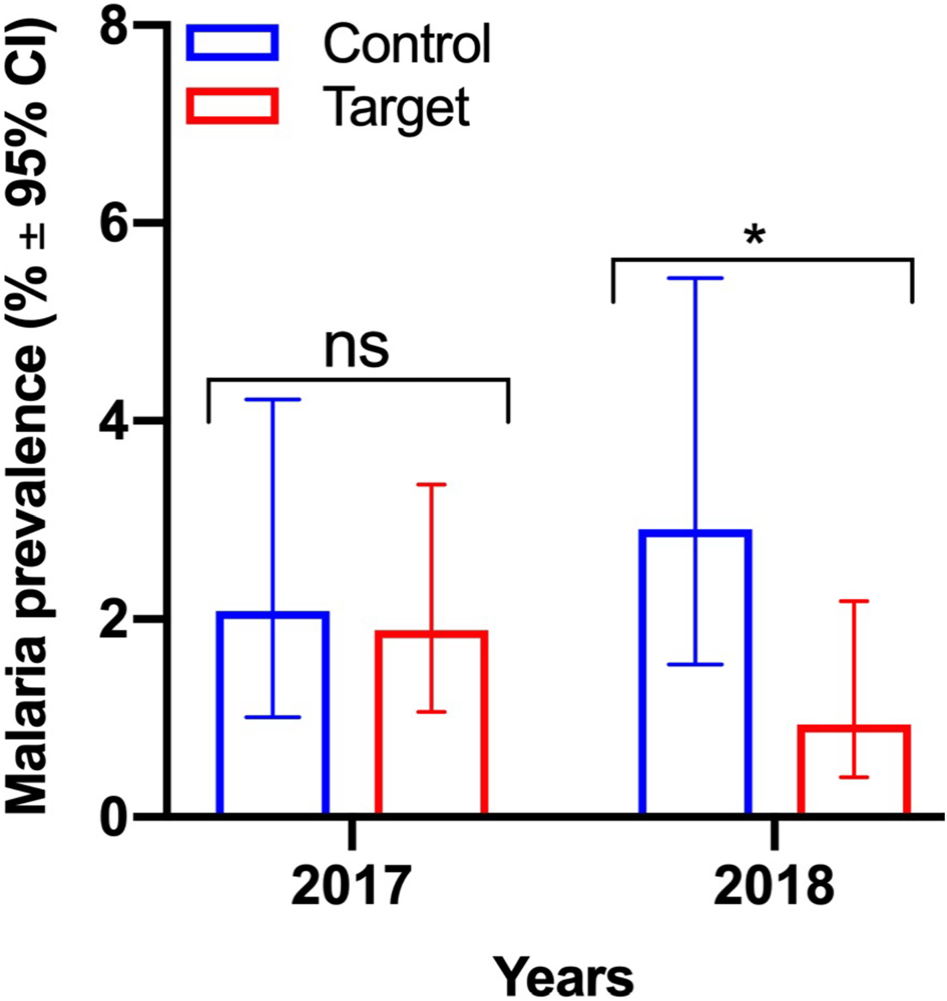
Malaria prevalence in the target and control villages of *Anopheles* reproductive swarm trapping. The target villages are: Chamoi, Dampha kunda, Tambasansang and Madina Yoro; and control ones are: Bakadagy and Mamasutu. The bars corresponds to the malaria prevalence ± 95% Confident Interval in 2017 and 2018. The significance of the difference is indicated (ns, p > 0.05; *p < 0.05).

In 2017, malaria prevalence ranged between 1.24 and 2.33%, and was similar between intervention (1.88%) and control villages (2.07%) (OR: 0.91; 95% CI 0.35–2.37, p = 0.849), (Fig. 4 and Table 3). In 2018, malaria prevalence ranged between 0 and 3.87% and was significantly lower in intervention (0.93%) than in control villages (2.91%) (OR: 0.32, 95% CI 0.11–0.97, p = 0.044, Fig. 4, Table 3 and Table S5).

**Table 3:** Unadjusted Odd Ratio from logistic regression analysis of malaria prevalence in target and control arms.

Villages	Type	Years of malariometric survey						Unadjusted odd ratio	
		2017			2018			OR (95% CI), reference: control	
		N	Positive slides	Prevalence (95% CI)	N	Positive slides	Prevalence (95% CI)	2017	2018
Chamoi	Target	152	3	2.01 (0.69–5.75)	129	2	1.55 (0.43–5.48)	OR = 0.91 (0.35, 2.37) p-value = 0.849	OR = 0.32 (0.11, 0.97) p-value = 0.044
Dampha Kunda		161	2	1.24 (0.34–4.42)	160	1	0.63 (0.11–3.45)		
Madina Yoro		85	2	2.33 (0.64–8.09)	87	0	0		
Tambasansang		185	4	2.16 (0.84–5.43)	156	2	1.28 (0.35–4.55)		
Bakadagy	Control	148	4	2.70 (1.06–6.74)	155	6	3.87 (1.79–8.19)		
Mamasutu		189	3	1.59 (0.54–4.56)	154	3	1.95 (0.66–5.57)		

## Discussion

Mass trapping of *Anopheles gambiae* males during swarming decreased significantly both indoor resting vector density and prevalence of malaria infection in intervention villages, although it did not have any effect on other entomological parameters, i.e., sporozoite rate and EIR. This is not surprising as indoor resting density should decrease by at least 80% for a significant effect on EIR^18,19^. An effect on indoor resting density of similar magnitude was also observed for other mosquito control tools such as solar mosquito trap^20^ and attractive toxic sugar baits (ATSB)^21^. Our primary goal was to investigate non-chemical strategies that could be effective for vector control, namely trapping swarming males with insect sweep nets rather than treating them with insecticides as previously described^16^. Despite no available data comparing both approaches, their effectiveness is probably similar as far targeted Anopheles gambiae populations are sensitive to the insecticide used.

Trapping *Anopheles* swarms has never been implemented as a vector control intervention although the contribution of male mosquitoes to higher indoor resting density and transmission intensity has been recently reported^17,22^, suggesting this approach has the potential of becoming a vector control tool^15,16^. In addition, this is an intervention that could be implemented with limited resources. Local volunteers were easily trained to collect swarm after sunset, at a time when they would have finished their usual daily activities such as farming or schooling. They carried out their task enthusiastically and reliably. A similar approach was taken for the control of larval habitats in Tanzania^23^. Despite a limitation to knowing the exhaustivity of swarming event numbers happening in the village, we can ensure that most swarms were identified in this pilot study accordingly to our experience in Anopheles’ reproductive biology study.

Swarms characteristics were investigated prior to swarms trapping. Indeed, knowing the vectors’ biological characteristics is essential to determine the place and the timing for the implementation of new vector control tools. Swarming of *Anopheles gambiae s.l.* began a few minutes after sunset, as also observed in Benin, Burkina Faso, Mali and Tanzania^15^. Despite reports of *Anopheles gambiae s.l.* indoor swarming in Tanzania^24^, all swarms in this study were outdoor, in open areas and close to human habitations. Moreover, swarm’s height and duration were similar to those described in other studies, although they were of smaller size than in Burkina Faso and Mali^15,22,25,26^. The association between indoor resting density and swarm size has already been reported in other countries, e.g., Benin^14^; small swarm size may be related with low indoor resting density oberved in the study sites.

Although only 5 out of the 23 swarms identified were *An. arabiensis*, this species represented about a third of all vectors collected by HLC, probably reflecting the increasing abundance of this species in eastern Gambia^27^. This may suggest that *An. arabiensis* swarms were missed, possibly because they are usually at a higher height from the ground^25^. The efficiency of swarm trapping may vary by species, with higher yield for *An. coluzzii* and *An. gambiae s.s.*. than for *An. arabiensis*.

Study villages were not randomized to either intervention or control arm, and this is a major limitation given that the two study groups, control and intervention, were more than 20 km apart and thus not necessarily comparable. Although the year prior to the intervention, malaria prevalence and the entomological parameters were similar between intervention and control villages, other factors could have been responsible of the intervention’s observed effect. For example, awareness on malaria may have increased because of the research team activity, resulting in prompt treatment seeking and/or increased use of ITN.

Malaria prevalence was determined by microscopy and thus a substantial proportion of low-density infections may have been missed. Nevertheless, microscopy diagnosed infections of higher parasite density and thus more transmissible to the vector^28^.

The low anopheline biting rates observed in 2017, at baseline, is probably due to the timing of the entomological collections as these started just after the peak of indoor resting density. In the intervention year, entomological collections started at the beginning of the transmission season, in July, explaining the higher number of mosquitoes collected by HLC. Nevertheless, the trend of indoor resting density in control villages was similar to that observed in 2017.

In conclusion, swarm trapping seems to be a promising, community-based vector control intervention that could reduce malaria prevalence by reducing vector density. Such results should be further investigated and confirmed by larger cluster-randomized trials.

## Materials and methods

The main goal of this study was to pilot mass swarm trapping as a potential vector control intervention. There were two objectives, first to describe swarming and mating behavior of malaria vectors and thus identify the swarm positions, and then determine the effect of mass swarm trapping on malaria transmission. In addition, all methods described below to achieve these objectives, were carried out in accordance with relevant guidelines and regulations.

### Study sites and design

The study was carried out in eastern Gambia, in Upper River Region (URR). Malaria transmission in The Gambia is seasonal and heterogeneous across the country, with relatively high prevalence in eastern Gambia^5^, despite high coverage of control interventions^6^. Six villages were identified and baseline data (swarming behaviour, *Anopheles gambiae s.l.* density and malaria prevalence) were collected in 2017. Mass swarm trapping was implemented in 2018 in four villages (Chamoi, Dampha Kunda, Tambasansang, and Madina Yoro) while the other two villages (Mamasutu and Bakadagy), about 20 km from the intervention villages to limit contamination^29^, were taken as controls (Fig. 1).

### *Anopheles gambiae s.l.* reproductive swarm characterization

Each study village was divided into several areas. Possible *An. gambiae s.l.* swarm markers (locations with high chances to find a swarm) were identified at daytime by trained volunteers (see Table 1) who went back in the evening to actively search for swarming events. They looked towards the lightest part of the sky, from ground level to about 4 m above the swarm markers. Swarms were confirmed by field supervisors and their duration and height above the ground was recorded. Swarms were sampled once, 5–10 min after their formation, using a standardized insect sweep net (120 cm diameter attached to a 1–1.5 m long stick, depending on the swarming height)^14^. The swarm location (latitude and longitude) was mapped within 2 m accuracy on background data using the Global Positioning System (GPS-Gamin^®^). Swarms were collected in the same locations for six evenings. Mosquitoes were transferred into cups, knocked down in a freezer, identified morphologically^30^ as belonging to *Anopheles gambiae s.l.*, counted and kept in silica gel in 1.5 ml Eppendorf tubes for further molecular identification.

### Swarms trapping as an intervention

The intervention (collection of swarms) was implemented between July and December 2018, covering the whole malaria transmission season. Trained local volunteers in intervention villages collected with a large insect net *Anopheles gambiae s.l.* males attending the swarming event for two consecutive days per week, at the same positions previously identified (Fig. 1). Collected mosquitoes were starved to death, morphologically confirmed as *An. gambiae s.l.*^30^, counted, and stored individually in 1.5 ml Eppendorf tube with silica gel at − 20 °C until DNA extraction.

### Entomological catches

#### Human landing catches (HLC)

HLC (indoors and outdoors) were carried out in all villages, from 8.00 p.m. to 7.00 a.m, in three locations per village, and for one night per survey (i.e., six person-night per village per survey). Surveys were carried out 6 weeks apart. There were 2 surveys in 2017 (September–December), and 4 surveys (July–December) in 2018. The collection teams were rotated between collection points on different nights to minimize sampling bias.

#### Pyrethrum spray catches for indoor resting collection (PSC)

PSC were carried out monthly, from September to December in 2017 and from July to December in 2018, in ten randomly selected houses per village, and two rooms per house^14^. Indoor resting density per house was estimated as the average number of malaria vectors per house, by month and village.

### Identification of *Anopheles gambiae* species complex

DNA was extracted from head/thoraces with Qiagen QIAxtractor robot according to the manufacturer’s protocol. Species-specific genotyping was performed^31^ and form-specific restriction enzyme digestion used to distinguish between *An. arabiensis* (292 bp), *An. gambiae s.s* (110 and 257 bp), *An. coluzzii* (367 bp)*, An. melas* (435 bp) and hybrid coluzzii/gambiae (110, 257 and 367 bp)^32^.

### Screening for *Plasmodium* sporozoites

DNAs extracted from mosquitoes’ head/thoraces (collected by HLC) was analysed using TaqMan SNP genotyping protocol^33^ to detect sporozoites of *Plasmodium falciparum*, *P. ovale*, *P. malariae,* and *P. vivax*.

### Malariometric survey

A cross-sectional survey was carried out at peak transmission season (November) for 2 consecutive years (2017 and 2018). In each villages, 150 individuals at least 6 months old and no history of travel within the previous month were randomly selected. After obtaining a written informed consent, a blood sample for microscopy was collected by finger-prick. Thick blood films were stained with 2% Giemsa for 30 min and examined by two independent microscopists. A third reader resolved any discrepancy. Blood smears were considered negative after reading 100 high power fields. Patients with clinical malaria (history of fever in the previous 24 h and/or body temperature ≥ 37.5 °C with a positive Rapid Diagnostic Test) were treated with artemether-lumefantrine, the first line treatment in The Gambia. The sample size was estimated on the assumption malaria prevalence would be 10%^6^; with 150 individuals per village, prevalence would be estimated with a precision of ± 5%.

### Ethical approval and consent

The study was approved by the Scientific Coordinating Committee of the Medical Research Council Unit The Gambia (SCC 1548) and the Gambia Government/MRC Joint Ethics Committee. The study team obtained verbal consent from the study communities at village meetings before field activities. Written informed consent was obtained from all individuals aged ≥ 18 years; parents/guardians provided written consent for children (< 18 years of age); assent was obtained from children aged 12–17 years. All household selected for entomological collection (using HLC and PSC) and volunteers for HLCs and swarm collection provided additional written informed consent.

### Data collection and statistical analyses

Data were collected using five different case report forms: swarm characterization, mass swarm trapping intervention, indoor resting densities assessment, mosquito sampling by HLC, and malariometric survey. Data were double entered into a Microsoft Excel datasheet, and the data supervisor reconciled the discrepancies via the verification process. *Anopheles* species morphological and molecular identification, sporozoites in salivary glands, and microscopy reading of thick blood films were extracted from the malaria diagnosis platforms into an Excel datasheet after double-checking and discrepancy reconciliation.

The indoor resting density was estimated as the mean of indoor resting *An. gambiae s.l.* females collected in ten houses per village. The biting rate was estimated by dividing the number of collected *An. gambiae s.l.* females by the number of volunteers and collection nights. The sporozoite rate was the proportion of *P. falciparum* positive mosquitoes divided by the total number of mosquitoes screened by PCR assay. The entomological inoculation rate (EIR) was estimated by multiplying the sporozoite rate by the biting rate^34^.

Swarm characteristics (swarm size, swarming duration and swarming height) were compared between Anopheles species (*An. gambiae s.s.*, *An. coluzzii* and *An. arabiensis*) using non-parametric Kruskal–Wallis test after confirming non-gaussian distribution with Shapiro–Wilk test. Indoor resting density, biting rate and EIR were compared between control and intervention villages using unadjusted negative binomial regression. The Z-test for the difference in two proportions was used to compare the sporozoite rate between control and intervention arms. Unadjusted logistic regression was used to compare malaria prevalence between control and intervention villages. Furthermore, the epidemiological and entomological endpoints of this study were estimated using village-level analysis and permutation tests to compare them between the two groups All statistical analyses were done with R^35^ (version 3.5.0), and the figures with Prism 9.

## Supplementary Information

Supplementary Information.

## Data Availability

Data supporting the conclusions of this article are included within the article. Raw data will be made available upon request to the corresponding author.
