# Supplementary material for: Anopheles gambiae s.l. swarms trapping as a complementary tool against residual malaria transmission in eastern Gambia
Source: Sci Rep. 2022 Oct 12;12:17057. doi: 10.1038/s41598-022-21577-7 (PMC9556655; doi:10.1038/s41598-022-21577-7)
Supplement: Supplementary file 1 — Supplementary Information. [file 41598_2022_21577_MOESM1_ESM.pdf]

Anopheles gambiae s.l. swarms trapping as a complementary tool against residual malaria transmission in eastern Gambia

Benoît Sessinou Assogba[1*], Salimina Sillah[1], Kevin O Opondo[1], Sheikh Tijan Cham[1], Muhammed M Camara[1], Lamin Jadama[1], Lamin Camara[1], Assane Ndiaye[4], Miriam Wathuo[2], Musa Jawara[1], Abdoulaye Diabaté[3], Jane Achan[1], Umberto D'Alessandro[1*].

[1]Disease Control and Elimination Theme, Medical Research Council , Unit The Gambia at London School of Hygiene and Tropical Medicine, PO Box273, Banjul, The Gambia;
[2]Statistic and Bioinformatic Department, Medical Research Council , Unit The Gambia at London School of Hygiene and Tropical Medicine, PO Box273, Banjul, The Gambia;
[3]Institut de Recherche en Science de la Santé/Centre Muraz, BP 545 Bobo-Dioulasso, Burkina Faso;
[4]Laboratoire d'Ecologie Vectorielle et Parasitaire, Faculté des Sciences et Techniques, Université Cheikh Anta Diop, Dakar, Sénégal.

*Corresponding author

Corresponding author:
Dr. Benoît Sessinou Assogba, Bsc, Msc, PhD
Research Fellow at Disease Control and Elimination Department,
Medical Research Council, Unit The Gambia at London School of Hygiene and Tropical Medicine, P O Box 273 Banjul, The Gambia, Tel: +2203214361
Email: Sessinou-Benoit.Assogba@lshtm.ac.uk

Supplementary Information

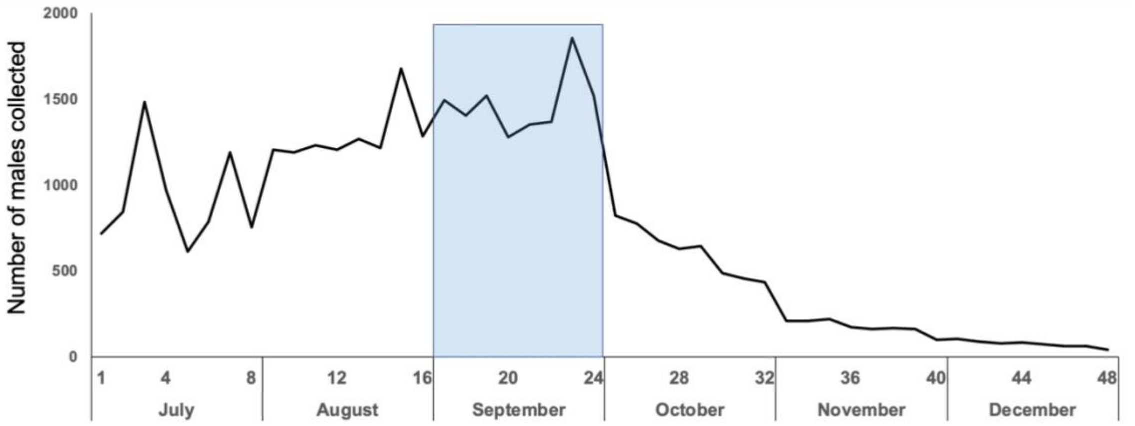

Figure S1: Number of *Anopheles gambiae s.l.* males collected by month. On X axis, the number correspond to the 1st collection to 48th one with two collection per week. July to December correspond the period of malaria season in The Gambia. The highest number of mosquitoes was collected in September which indicated with the light blue box

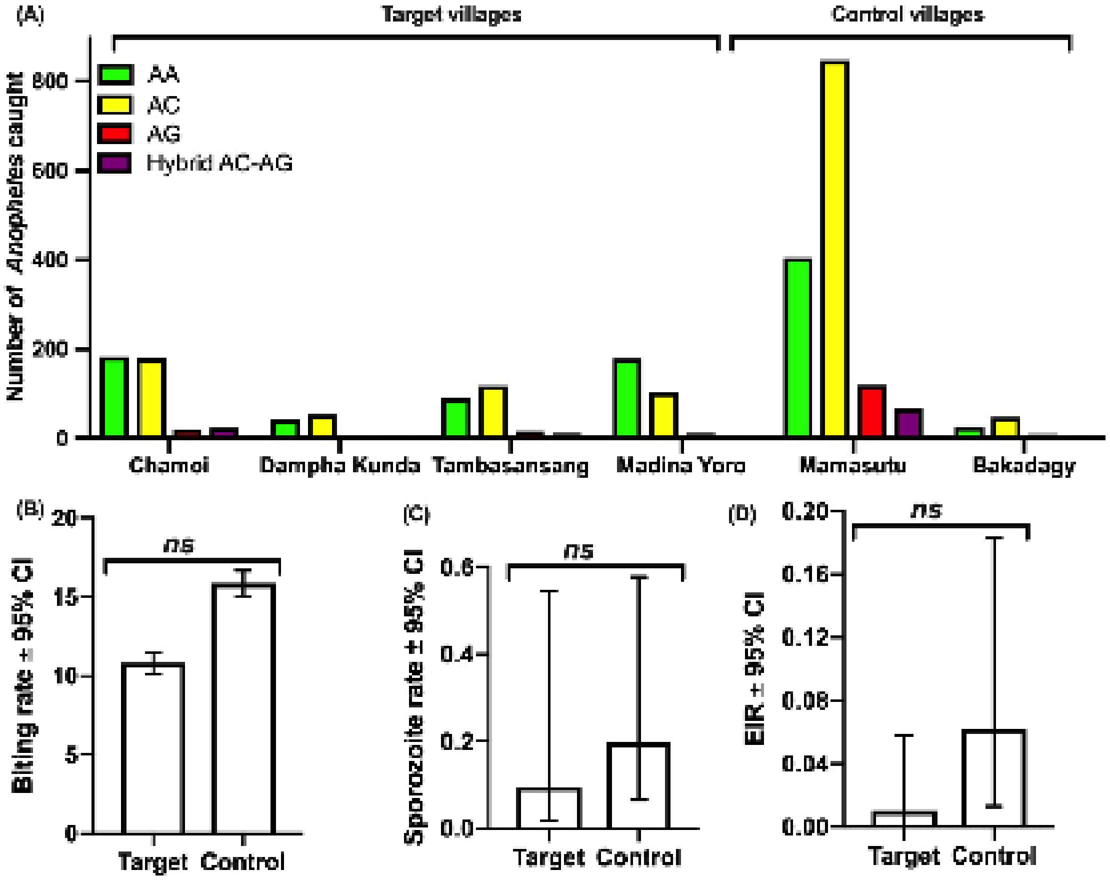

Figure S2. Biting rate, sporozoite rate and entomological inoculation rate (EIR) by study arm. (A) showing the distribution of *An. arabiensis, An. coluzzii, An. gambiae s.s.* and hybrid *An. coluzzii - gambiae s.s.* using Human landing catch (HLC) in both target and control villages. (B) Biting rate: the bar indicates the proportion of *An. gambiae s.l*. bite received per human volunteer and per night with 95% confident interval. (C) Sporozoite rate: the bar indicates the proportion of *An. gambiae s.l*. infected with Plasmodium falciparum amongst the malaria mosquito caught with 95% confident interval. (D) EIR: number of *An. gambiae s.l*. infected with *Plasmodium falciparum* bite received by per human volunteer and per year with 95% confident interval. “ns” indicate no significant different of biting rate, sporozoite rate and EIR between the target and control arms (p-value < 0.05).

Table S1: Effect of intervention on mosquito density by year following the cluster-level analysis

Year	Arm	Crude IRR (95% CI)	P-value
2017	Control	1	
	Intervention	0.99 (0.89, 1.10)	0.960
2018	Control	1	
	Intervention	0.58 (0.45, 0.75)	<0.001

Table S2: Effect of intervention on biting rate following the cluster-level analysis

Arm	Crude IRR (95% CI)	P-value
Control	1	
Intervention	0.66 (0.10, 4.40)	0.678

Table S3: Effect of intervention on Sporozoite rate following the cluster-level analysis

Arm	Crude OR (95% CI)	P-value
Control	1	
Intervention	0.81 (0.20, 3.29)	0.558

Table S4. Age and sex characteristics of participants involved in malariometric survey.

Years	Villages	Participants					
		N	Sex (% Female)	Child		Adult	
				%	Age Median (IQR)	%	Age Median (IQR)
2017	Chamoi	152	53.94	65.78	7.0 (3.0 - 11.5)	34.22	32.0 (26.0 - 49.5)
	Dampha Kunda	161	54.03	55.9	5.0 (3.0 - 8.0)	44.1	42.0 (30.0 - 57.5)
	Madina Yoro	85	58.82	45.88	7.0 (4.0 - 10.0)	54.12	38.0 (25.0 - 60.0)
	Tambasansang	186	54.3	66.12	8.0 (7.0 - 9.0)	33.88	47.0 (30.0 - 64.0)
	Bakadagy	148	52.02	65.54	8.0 (3.5 - 12.5)	34.46	30.5 (22.0 - 50.0)
	Mamasutu	189	51.32	43.91	7.0 (4.0 - 10.0)	56.09	33.0 (22.5 - 52.5)
2018	Chamoi	163	52.14	65.03	6.0 (4.0 - 9.0)	34.97	32.0 (24.0 - 48.0)
	Dampha Kunda	160	56.87	61.87	8.0 (5.0 - 10.0)	38.13	35.0 (26.0 - 51.0)
	Madina Yoro	93	54.83	50.53	7.0 (4.0 - 10.0)	49.47	40.5 (25.0 - 52.0)
	Tambasansang	163	55.21	55.82	4.0 (3.0 - 6.0)	44.18	39.0 (27.0 - 52.5)
	Bakadagy	155	50.96	61.93	6.0 (4.0 - 8.0)	38.07	38.0 (24.0 - 58.0)
	Mamasutu	158	55.06	62.65	5.0 (3.0 - 8.0)	37.35	28.0 (23.0 - 42.0)

N correspond to the number of study participants in each village. % correspond to the percentage either of children or adults out of total participants. IQR corresponds to interquartile range of age median.

Table S5: Effect of intervention on malaria prevalence by year following the cluster-level analysis

Year	Arm	Crude OR (95% CI)	P-value
2017	Control	1	
	Intervention	0.94 (0.44, 2.04)	0.819
2018	Control	1	
	Intervention	0.38 (0.13, 1.14)	0.058
